# Implementing Pulse Oximetry Screening for Critical Congenital Heart Disease in São Tomé and Príncipe: Lessons from a Pilot Programme

**DOI:** 10.5334/gh.1599

**Published:** 2026-11-12

**Authors:** Sara C. Pereira, Miryan Cassandra, Swasilanne Bandeira, Daniel Carvalho, António Pires, Thierry E. Mertens, Ana Abecasis

**Affiliations:** 1 Global Health and Tropical Medicine, GHTM, LA-REAL, Instituto de Higiene e Medicina Tropical, IHMT, Universidade NOVA de Lisboa, Lisboa, Portugal; 2 Cardiology Department, Hospital Ayres de Menezes, São Tomé, São Toméand Príncipe; 3 Paediatric Department, Hospital Ayres de Menezes, São Tomé, São Toméand Príncipe; 4 Paediatric Cardiology Department and Referral Centre for Congenital Heart Disease, University of Coimbra, Coimbra, Portugal

**Keywords:** Congenital Heart Disease, Pulse Oximetry, Newborn Screening, Low- and Middle-Income Countries, Implementation, Health Systems Strengthening

## Abstract

Pulse oximetry (POX) screening improves early detection of critical congenital heart disease (CCHD) but has rarely been implemented in newborn care in sub-Saharan Africa. We introduced POX screening at São Tomé and Príncipe’s main maternity unit, integrating staff training and a simplified screening algorithm. Six CCHD diagnoses were documented at the maternity unit after positive screens, two in already symptomatic infants. Screening coverage is unknown and screen-negative infants were not systematically followed up, so sensitivity cannot be determined and missed cases cannot be excluded. Staff interviews identified workforce shortages, equipment limitations, difficulties interpreting the algorithm, caregiver reluctance to delay discharge, and inconsistent documentation. Training was reinforced, caregiver materials and wrap-type neonatal probes were introduced, and the maternity informatic system was adapted. POX screening was delivered within routine care, but reliable documentation and post-discharge follow-up are needed before coverage or accuracy can be assessed.

## Graphic Abstract

**Figure:**
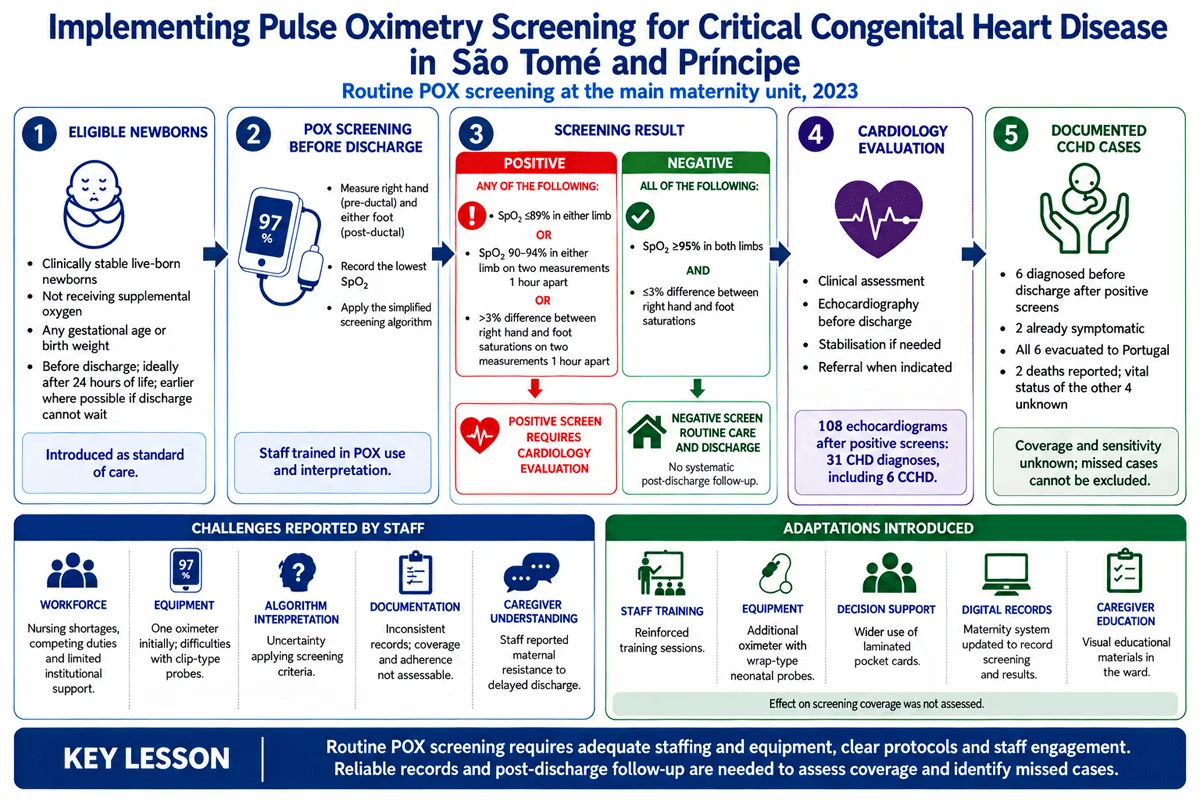


## Background

Congenital heart disease (CHD) is the most common congenital anomaly worldwide, with an estimated prevalence of 8–9.5 per 1,000 live births (1,2). It is the leading cause of birth defect-related illness and death in infants, accounting for up to 10% of infant mortality associated with congenital anomalies, particularly in low- and middle-income countries (LMICs), where delayed diagnosis and limited access to specialised care remain common (3,4,5). Critical congenital heart disease (CCHD), a subset of CHD requiring surgical or catheter-based intervention within the first 28 days of life, accounts for approximately 25% of CHD cases (6,7).

Delayed diagnosis of CCHD is associated with increased neonatal morbidity and mortality, emergency admissions, and poorer surgical outcomes (6,8). Early identification through pulse oximetry (POX) screening enables timely cardiology evaluation, stabilisation, and access to specialised care before clinical deterioration. Although POX screening is widely used in high-income countries, evidence from routine implementation in sub-Saharan African settings remains limited (5,8,9).

São Tomé and Príncipe is a lower-middle-income Small Island Developing State in the Gulf of Guinea with limited diagnostic capacity for CHD. Prenatal ultrasound is constrained by shortages of trained personnel and equipment, and children requiring cardiac surgery are referred overseas, mainly to Portugal, often after delayed diagnosis. In 2023, CHD accounted for 15% of paediatric medical evacuations.

A pilot POX screening programme was introduced at Hospital Ayres de Menezes. We describe its implementation, operational challenges, adaptations and lessons for other resource-limited settings.

## Method

POX screening for CCHD was introduced between January and December 2023 at Hospital Ayres de Menezes, São Tomé and Príncipe’s main maternity unit, as part of routine neonatal care. All clinically stable live-born newborns who were not receiving supplemental oxygen were eligible, irrespective of gestational age or birth weight; no other formal exclusion criteria were documented. Screening targeted newborns before discharge, ideally after 24 hours of life. Infants discharged earlier were screened before departure where possible. Fetal echocardiography is not routinely performed in São Tomé and Príncipe. POX screening was introduced as standard of care in collaboration with the cardiology department. A simplified algorithm adapted from international recommendations was used (Figure 1) (10). Screening was performed mainly by five newborn nursery nurses, with one on duty per shift. All 25 maternity nurses and two physicians attended one of four two-hour sessions led by the principal investigator to support implementation and staff rotations. Training covered CCHD, oximeter use, newborn preparation and positioning, timing, interpretation and referral; laminated pocket cards summarised the algorithm. Pre-ductal and post-ductal oxygen saturations were measured and interpreted immediately by the screener. Standardised newborn handling was taught; adherence was not measured reliably. One Gruppo Industriale Milanese Aghi S.p.A. (Gima) OXY-10 Finger Pulse Oximeter with clip-type probes was used initially; a second GIMA OXY-10 with wrap-type probes, added at the end of 2023, did not contribute to the reported screening. Positive screens prompted cardiology evaluation and echocardiography before discharge, using a GE Vivid IQ system, performed by one of two cardiologists or two cardiology technicians according to availability.

**Figure 1:**
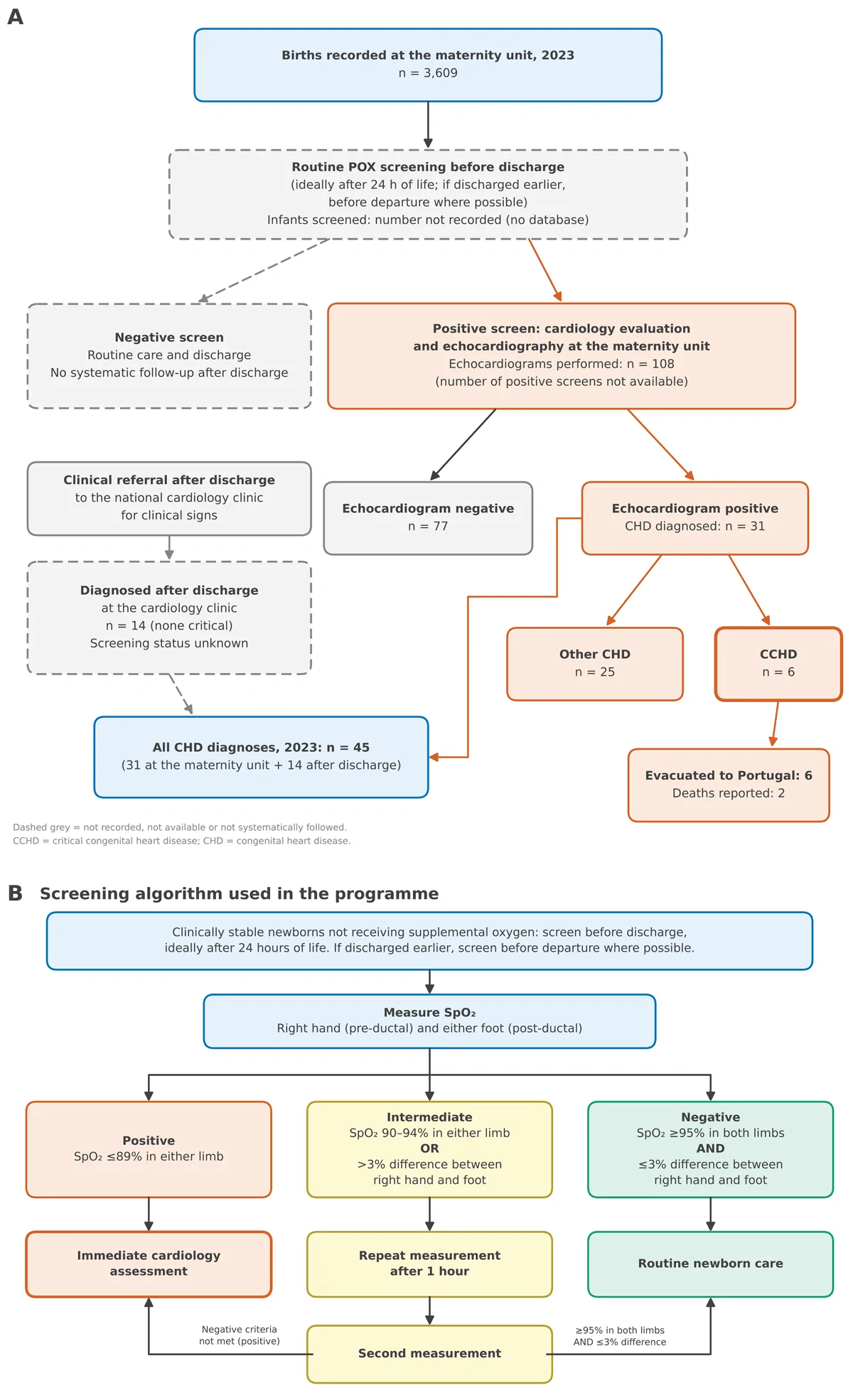
Flow of newborns, screening algorithm and echocardiography results in the pilot pulse oximetry screening programme for CCHD at Hospital Ayres de Menezes, São Tomé and Príncipe, 2023. Boxes show numbers of newborns or echocardiographic examinations, as indicated. Dashed grey boxes indicate quantities or outcomes that were not recorded or were unavailable: the number of newborns screened, the number of positive screens, and post-discharge outcomes among newborns with negative screens. Cardiology records documented 108 echocardiograms performed at the maternity unit, of which 31 identified CHD and 77 showed no CHD. Fourteen infants were diagnosed with CHD after discharge following referral to the national cardiology clinic for clinical signs; their screening status was unknown. Screening targeted newborns before discharge, ideally after 24 hours of life. Infants discharged earlier were screened before departure where possible. Panel B shows the screening algorithm used in the pilot programme. A positive screen was defined as any of the following: SpO_2_ <90% in either limb at any measurement; SpO_2_ 90–94% in either limb on two measurements 1 hour apart; or a difference of >3 percentage points between the right hand and foot on two measurements 1 hour apart. A negative screen required SpO_2_ ≥95% in both limbs and a difference of ≤3 percentage points. CCHD, critical congenital heart disease; CHD, congenital heart disease; POX, pulse oximetry; SpO_2_, oxygen saturation. Source: Figure prepared by the authors. Screening algorithm adapted from Martin et al. (10).

A documentation stamp in each child’s medical booklet recorded screening completion and results; entries were not compiled into a database, so no aggregate screening count was available. Implementation was evaluated using routine service data and semi-structured interviews with eight nurses and two physicians involved in screening covering time and staff availability, engaging mothers, technical execution, interpretation and coordination with cardiology. Interview notes were reviewed descriptively; no formal thematic analysis was performed, and mothers were not interviewed. The Ministry of Health and the hospital authorised the introduction of POX screening as standard of care and determined that ethics committee review and individual consent, including for staff interviews, were not required. No ethics committee review was obtained. Data were de-identified.

## Results

During the implementation period, 3,609 births were recorded, reflecting maternity activity rather than a screening denominator; the total numbers of newborns screened and positive screens were not available (Figure 1). Of 45 diagnoses of CHD, 31 were made at the maternity unit among 108 echocardiograms performed after positive screens (77 showed no CHD), and 14 after discharge, following referral to the national cardiology clinic for clinical signs; none of these 14 had CCHD, and their screening status was unknown.

All six CCHD cases (Table 1) were diagnosed by echocardiography at the maternity unit after positive screens; two infants were already symptomatic, so detection cannot be attributed to POX alone. All six were evacuated to Portugal; two deaths were reported (both in Portugal; timing and cause unknown), and the course and vital status of the other four were unavailable.

**Table 1:** Infants with critical congenital heart disease (CCHD) diagnosed during the implementation period (n = 6).


CHARACTERISTIC	INFANTS WITH CCHD (n = 6)

Share of all CHD diagnoses (n = 45)	6 (13.3%)

Screened with POX	6 of 6

Diagnosed at the maternity unit by echocardiography, among examinations performed after positive screens	6 of 6

Symptomatic when assessed	2 of 6 (hypoplastic right heart syndrome, n = 1; pulmonary atresia, n = 1)

Not reported as symptomatic	4 of 6

Recorded CCHD diagnoses	Pulmonary atresia, n = 3; double-outlet right ventricle with ventricular septal defect and pulmonary stenosis, n = 2; hypoplastic right heart syndrome, n = 1

Medically evacuated to Portugal for specialised treatment	6 of 6

Deaths reported	2 of 6: pulmonary atresia, n = 1; hypoplastic right heart syndrome, n = 1. Both deaths occurred in Portugal; timing and cause of death unknown.

Follow-up after evacuation	No follow-up information available for the other four infants; their vital status is unknown


CCHD, critical congenital heart disease; CHD, congenital heart disease; POX, pulse oximetry. Counts are aggregate figures supplied by the cardiology team; individual-level data were not available for this report. The percentage uses all 45 CHD diagnoses as the denominator. The six CCHD diagnoses were pulmonary atresia (n = 3), double-outlet right ventricle with ventricular septal defect and pulmonary stenosis (n = 2), and hypoplastic right heart syndrome (n = 1). All six infants were medically evacuated to Portugal. Two deaths were reported: one infant with pulmonary atresia and the infant with hypoplastic right heart syndrome. Both deaths occurred in Portugal; their timing and cause were unknown. No follow-up information was available for the other four infants, including their vital status. Information on interventions received after evacuation was not available for any of the six infants.

Staff interviews identified nursing shortages, competing clinical responsibilities, limited oximeter availability, technical difficulties with clip-type probes, uncertainty in interpreting the algorithm, and reluctance to assume additional responsibilities without institutional support as major barriers (Figure 2). Staff also reported maternal resistance to delayed discharge, which they related to limited understanding of screening.

**Figure 2:**
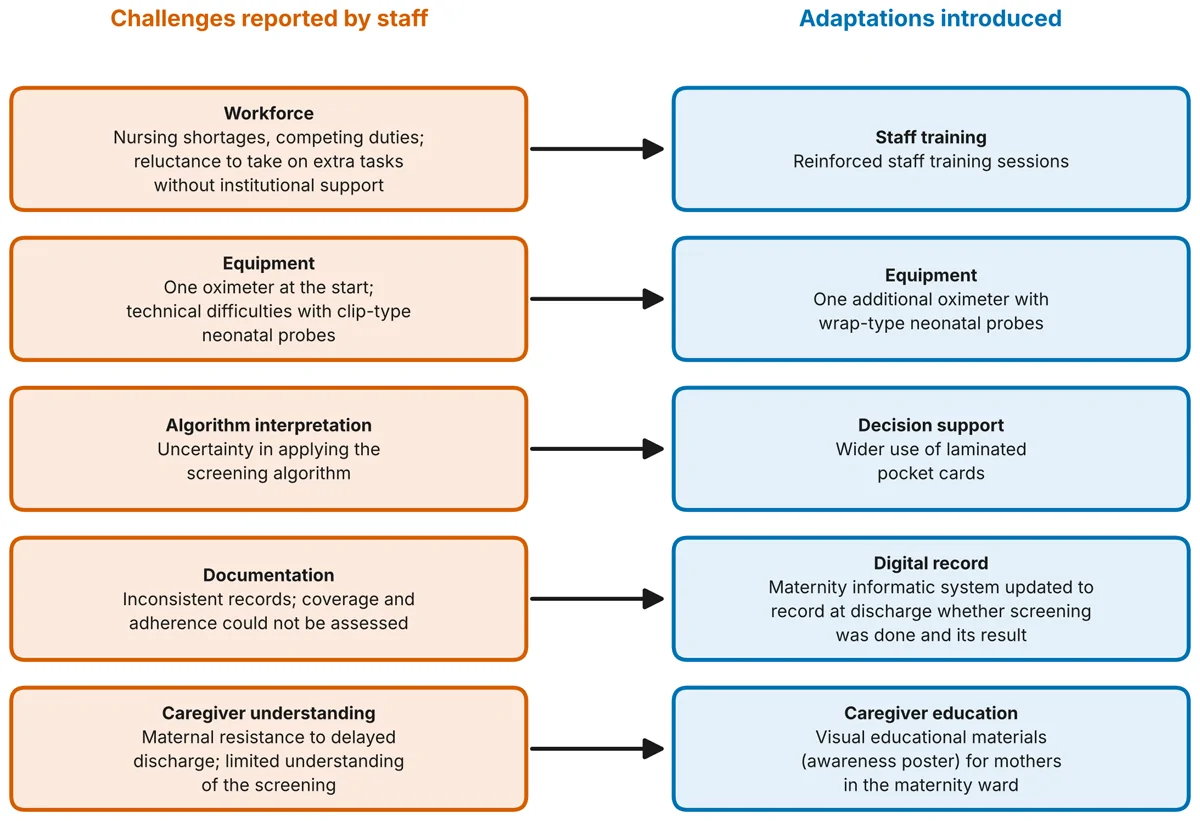
Implementation challenges reported by maternity staff in semi-structured interviews and adaptations introduced during the initial implementation of routine pulse oximetry screening at Hospital Ayres de Menezes, São Tomé and Príncipe, 2023. Source: Figure prepared by the authors.

Inconsistent documentation prevented reliable assessment of screening coverage and adherence. In response, training was reinforced, educational materials for caregivers were placed in the ward, an additional oximeter with wrap-type probes was procured, pocket card use was widened, and the maternity informatic system was updated to capture screening performance and results (Figure 2). Their effect on coverage was not assessed.

## Conclusions

POX screening was delivered within routine neonatal care at one maternity unit, but staffing, equipment, interpretation and monitoring constraints limited implementation. Staff-reported barriers suggest that adequate staffing and equipment, simplified protocols, reliable documentation and staff engagement are important.

Screening coverage is unknown and screen-negative infants were not systematically followed up, so sensitivity and false-negative results cannot be determined and missed cases cannot be excluded; undiagnosed infants who died would not have been captured. Outcomes after evacuation were largely unknown, lesion-level criteria for non-critical CHD were not specified and echocardiograms were not independently reviewed (so the 45 diagnoses do not measure diagnostic accuracy), and the single-site design and absence of ethics committee review limit interpretation. These findings do not establish coverage, diagnostic accuracy, improved survival or readiness for national scale-up.

Future implementation should include routine recording of screening, structured post-discharge follow-up and linkage with diagnostic and outcome records to identify missed cases.
